# Effect of radical lymphadenectomy in colorectal cancer with para-aortic lymph node metastasis: a systematic review and meta-analysis

**DOI:** 10.1186/s12893-022-01631-x

**Published:** 2022-05-14

**Authors:** Pengyue Zhao, Xingpeng Yang, Yang Yan, Jiaqi Yang, Songyan Li, Xiaohui Du

**Affiliations:** Department of General Surgery, First Medical Center of Chinese, PLA General Hospital, 28 Fuxing Road, Haidian, Beijing, 100853 People’s Republic of China

**Keywords:** Colorectal cancer, PALNM, Survival, Complication, Meta-analysis

## Abstract

**Background:**

Colorectal cancer (CRC) with para-aortic lymph node metastasis (PALNM) is an intractable clinical situation, and the role of radical lymphadenectomy in the treatment of CRC with PALNM is still controversial. The aim of the current system review and meta-analysis is to evaluate the clinical efficacy and safety of radical lymphadenectomy in CRC patients with PALAN.

**Methods:**

We performed a systematic search of PubMed, Embase, Cochrane Library and other online databases up to 31 October 2021. The clinical data including overall survival and postoperative complications were screened and analyzed after data extraction. Odds ratios (ORs) were applied to analyze these dichotomous outcomes with a fixed effects model.

**Results:**

A total of 7 available retrospective clinical studies involving 327 patients were finally included. CRC patients with PALNM who underwent radical lymphadenectomy showed significantly overall survival (OR: 6.80, 95% CI: 3.46–13.38, P < 0.01; I^2^ = 0%) when compared to those who did not receive radical lymphadenectomy. Moreover, in terms of postoperative complications (OR: 0.71, 95% CI: 0.35–1.44, P = 0.48; I^2^ = 0%), there was no statistical difference between radical lymphadenectomy treatment and control groups.

**Conclusions:**

The radical lymphadenectomy treatment has showed the expected clinical efficacy in prolonging overall survival time of CRC patients with PALAN. Moreover, the preemptive radical lymphadenectomy could not cause additional postoperative complications.

**Supplementary Information:**

The online version contains supplementary material available at 10.1186/s12893-022-01631-x.

## Background

Colorectal cancer (CRC) ranks third and second in morbidity and mortality, respectively [1], which is a heavy burden on global health and medical services. Approximately 50% of CRC patients will suffer distant metastasis at a certain time in the course of the disease [2]. Once this occurs, the prognosis of patients will be significantly deteriorated. Surgical intervention is still considered to be the most effective way to cure CRC patients with metastasis. Therefore, resection of the primary and metastatic lesions becomes the standard surgical procedure to improve the prognosis of CRC patients with metastasis [3, 4]. However, the management of CRC patients with para-aortic lymph node metastasis (PALNM) remains controversial in the gastrointestinal surgical field for the tricky definition of these patients’ clinical stage. In 2019, the Japanese Society for Cancer of the Colon and Rectum (JSCCR) adjusted the staging of PALNM to four stages, which is consistent with the eighth edition of the Joint American Council on Cancer (AJCC) cancer staging, implying a constant updating and evolution of surgeons’ perceptions [5–7].

The controversy regarding PALMN is mainly focused on the necessity and feasibility of the surgical resection, although there have been numerous studies with positive results [8, 9]. AJCC considers that PALNM represents disseminated metastasis and should be classified as stage IV disease. While Japanese experts tended to classify PALNM as a regional, stage III disease. As for China, there were no unified diagnosis and treatment standard or expert guidelines for PALNM treatment so far. Given such various views on the management of PALNM, no wonder there are controversies on the choice of the optimal treatment approach for CRC patients with PALNM. Currently, the need for radical lymphadenectomy in CRC patients with PALNM largely depends on the surgeons’ occupational preference. To the best of our knowledge, no randomized controlled trials (RCTs) have been reported on CRC patients with PALNM worldwide. Moreover, although a number of cohort studies and case–control studies have been published with promising conclusions regarding the management of PALNM, the clinical characteristics of these studies, such as retrospective and small sample sizes, greatly limited their level of evidence-based medicine. In view of the controversy over treatment strategies about PALNM, it is particularly significant to conduct a systematic analysis of existing studies to form a relatively unified guidance scheme and provide professional reference for gastrointestinal surgeons.

## Methods

### Search strategy

A comprehensive literature search of PubMed, Embase and Cochrane Library irrespective of languages was conducted for research related to the management of CRC with PALNM up to 31 October 2021. We conceived a strategy that combined exploded medical subject heading (MeSH) terms and entry terms, and the terms were as follows: “Colorectal Neoplasms”, “Colorectal Neoplasm”, “Neoplasm, Colorectal”, “Colorectal Carcinoma”, “Carcinoma, Colorectal”, “Carcinomas, Colorectal”, “Colorectal Carcinomas”, “Colorectal Cancer”, “Cancer, Colorectal”, “Cancers, Colorectal”, “Colorectal Cancers”, “Colorectal Tumors”, “Colorectal Tumor”, “Tumor, Colorectal”, “Tumors, Colorectal”, “Neoplasms, Colorectal”, “Lymphatic Metastases”, “Lymph Node Metastasis”, “Lymph Node Metastases”, “Metastasis, Lymph Node”, “para-aortic” and “paraaortic”. Meanwhile, we identified and included some studies by screening the reference lists of similar reviews or systematic reviews. The current study was conducted in conformity to the Preferred Reporting Items for Systematic Reviews and Meta-Analysis (PRISMA) statements [10].

### Inclusion and exclusion criteria

The inclusion criteria were as follows: (1) the studies comparing the efficacy and safety of radical lymphadenectomy with those not undergoing lymphadenectomy or radical lymphadenectomy among adult CRC patients with PALNM; (2) all included patients in the study must have clear preoperative imaging data and postoperative pathology reports to confirm PALNM.

The exclusion criteria included: (1) CRC patients with distant organs metastases; (2) CRC patients with other non-regional lymph nodes metastases rather than PALNM; (3) the studies had no control groups; (4) ongoing clinical trials; (5) lack of sufficient information or without follow-up.

Two reviewers independently screened and identified all potentially included studies. In the process of inclusion and exclusion, titles and abstracts were first checked, any conflicts between two reviewers were resolved by the third reviewer to achieve consensus. When the screening process of titles and abstracts was finished, full-text was subsequently assessed to determine its eligibility.

### Data extraction and quality evaluation

The data from all eligible studies was independently extracted by reviewers with a standardized and predesigned table. The characteristics of eligible studies including first author, publication year, study type, total number of enrolled patients, intervention and comparison, primary outcomes etc. were recorded. Moreover, the inconsistency of extracted data was resolved by discussion or consulting another reviewer until a consensus was achieved.

We also assessed the risk of bias of each eligible study according to its study type. The quality of observational study (cohort study and case–control study) was judged using the Risk of Bias in Non-randomized Studies—of Interventions (ROBINS-I) tool. The ROBINS-I tool assesses bias across seven domains including: confounding, selection of participants, classification of interventions, deviations from intended interventions, missing data, measurement of outcomes and selection of reported results. For each domain an outcome of low, moderate, serious, critical and no information for risk of bias is recorded. An overall risk of bias judgement is then determined through combination of the above seven domains [11].

### Outcome measurements

Considering the generalization of overall survival (OS) in determining the prognosis of cancer patients, we chose OS as primary endpoint regardless of the follow-up time. For research purposes, 3-year or 5-year OS was acceptable. Besides, OS was preferentially reported by majority of included studies. The secondary endpoint was the rate of adverse reactions, which could reflect the safety of treatment. In addition, we performed a sensitivity analysis that excluded every study, each at a given time, to prove whether our conclusions were stable [12].

### Statistical analysis

Review Manager (RevMan 5.3, Copenhagen: the Nordic Cochrane Center, the Cochrane Collaboration, 2014) was used to conduct the current Meta-analysis. Odds ratios (ORs) were applied for dichotomous outcomes, and pooled proportions were calculated with 95% confidence intervals (95% CI). The heterogeneity of each outcome was evaluated by calculating the *I*^2^ statistic. The I^2^ > 50% indicated a significant heterogeneity and random effects model was applied, otherwise fixed effects model was used accordingly. In addition, we constructed the Funnel plot that could be visually inspected to assess the publication bias. Meanwhile, both Begg’s and Egger’s tests were conducted and a two-sided *P*-value < 0.05 was considered as statistically significance. In order to seek potential factors that could influence the heterogeneity, we performed subgroup analysis according to various subgroup standards, which was conductive to validating the consistency and robustness of our finding. To be specific, all eligible clinical studies were stratified by type of study (cohort study or case–control study), management (dissection or R0 resection), timing of metastasis (synchronous or metachronous), publication country (Korea or Japan) and location of primary tumors (left-sided or all).

## Results

### Literature search and characteristics of included studies

**Fig. 1:**
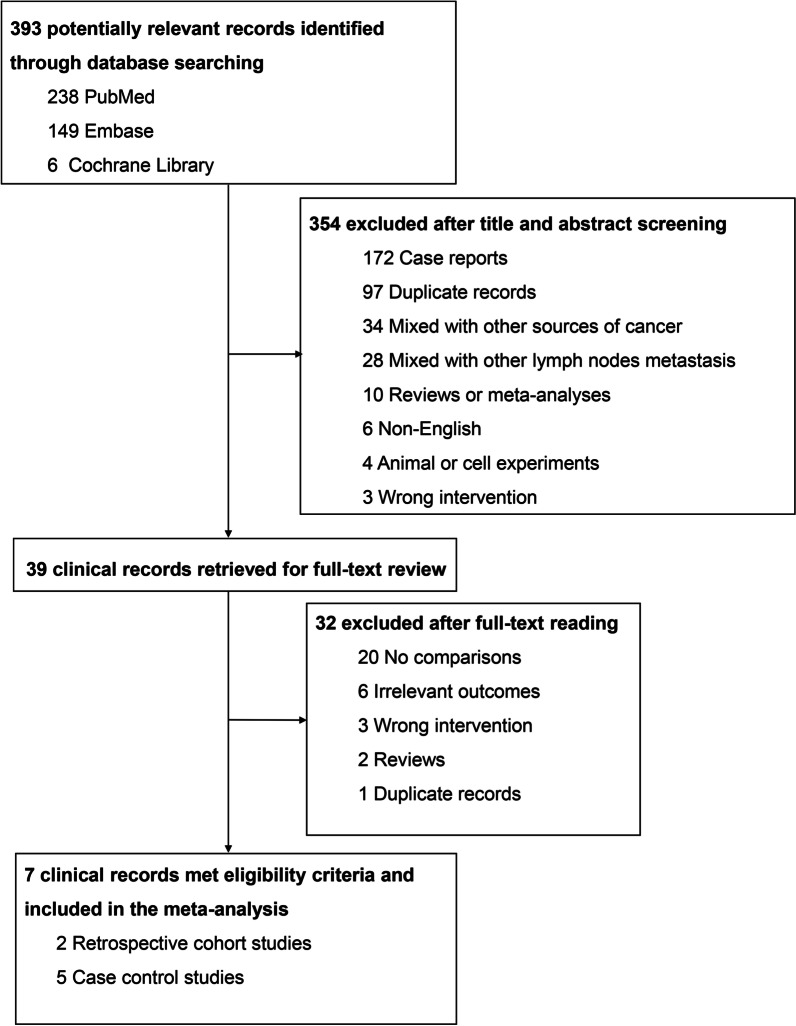
Flowchart for selection process

The database search initially identified 393 potentially relevant records (238 from PubMed, 149 from Embase and 6 from Cochrane Library). Through checking titles and abstracts, 354 literature was excluded for the reason of case reports, duplication, mixing with other sources of cancer, mixing with other lymph nodes metastasis, reviews or meta-analyses, non-English, animal or cell experiments and wrong intervention. The remaining 39 records were further determined for eligibility by reviewing the full text, and 32 of them were eliminated on account of no comparison, irrelevant outcomes, wrong intervention, duplication etc. Eventually, 7 clinical studies were included in our final meta-analysis. It’s worth noting that all eligible studies were retrospective cohort or case control series. The detail screening process was shown in Fig. 1.

Table 1 presented the characteristics of all eligible studies in detail. 7 retrospective clinical studies (5 case control and 2 cohort studies) including 327 patients were enrolled in current research. Among all included studies, the treatment of the experimental group was dissection, R0 resection and radical resection. Three studies adopted the management of non-dissection for PALNM in control groups, and the rest 4 implemented R1 or R2 resection. In addition, the majority of studies chose OS as the primary outcome, while Ichikawa’s selected 3-year recurrence‐free survival (RFS) as the first endpoint. In terms of the metastasis time, there were 2 and 1 studies on simultaneous and metachronous metastasis, respectively. Three studies covered patients with both types of metastases, and one study did not clearly specify the status of metastasis.

**Table 1:** Characteristic of included clinical studies

Study	Types of studies	NO. of patients	Intervention		Primary endpoint	Synchronous or Metachronous	Country	Location of tumor
			Treatment group	Comparison group				
Lee, S. C. et al., 2021	CCS	73	Dissection	Non-dissection	5-year OS	Synchronous	Korea	All
Lee, J. et al., 2021	CS	19	R0 resection	R2 resection	5-year OS	No instructions	Korea	Left-sided
Ichikawa, Y. et al., 2021	CS	28	Radical resections	Targeted dissections	3-year RFS	Both (16:12)	Japan	All
Sahara, K. et al., 2019	CCS	62	R0 resection	R1/R2 resection	5-year OS	Synchronous	Japan	Left-sided
Nakai, N. et al., 2017	CCS	30	R0 resection	R1/R2 resection	5-year OS	Both (16:14)	Japan	Left-sided
Choi, P. W. et al., 2010	CCS	77	Dissection	Non-dissection	5-year OS	Both (19:5)	Korea	All
Byung, S. M. et al., 2008	CCS	38	Dissection	Non-dissection	5-year OS	Metachronous	Korea	All

*CS* cohort study, *CCS* case–control study, *OS* overall survival, *RFS* recurrence‐free survival, *NOS* the Newcastle–Ottawa Scale

### Primary outcome: overall survival

**Fig. 2:**
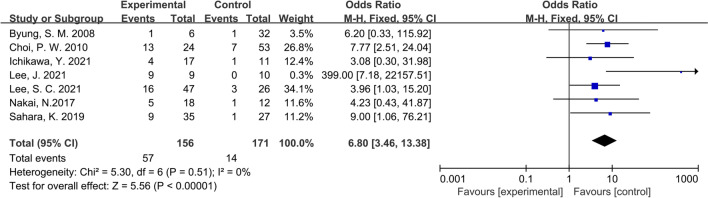
Forest plot of OS comparing experimental group to control group among CRC patients with PALNM

All the included studies, except Ichikawa’ s, chose OS as the primary clinical outcome and were followed up for 5 years. Compared to those who did not receive lymphadenectomy or underwent non-R0 resection, CRC patients with PALNM that received radical lymphadenectomy had significant survival time benefit (OR: 6.80, 95% CI: 3.46–13.38, P < 0.01; I^2^ = 0%) (Fig. 2). Through performing a sensitivity analysis that excluded every study, each at a time, we found that the conclusion was proven to be stable.

### Rate of postoperative adverse reactions

**Fig. 3:**
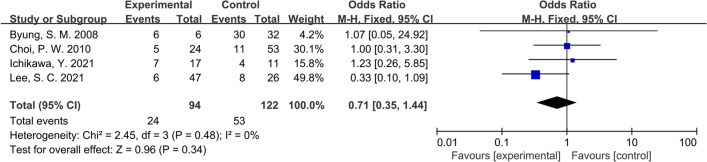
Forest plot of rate of postoperative adverse reactions comparing experimental group to control group among CRC patients with PALNM

Of all the articles included in this study, four of them compared the incidence of postoperative complications between patients who underwent radical lymphadenectomy and those did not. As presented in Fig. 3, there was no significant difference in the rate of postoperative adverse reactions between the two groups (OR: 0.71, 95% CI: 0.35–1.44, P = 0.48; I^2^ = 0%). Similarly, this conclusion was quite stable after conducting the sensitivity analysis.

### Subgroup analysis

**Fig. 4:**
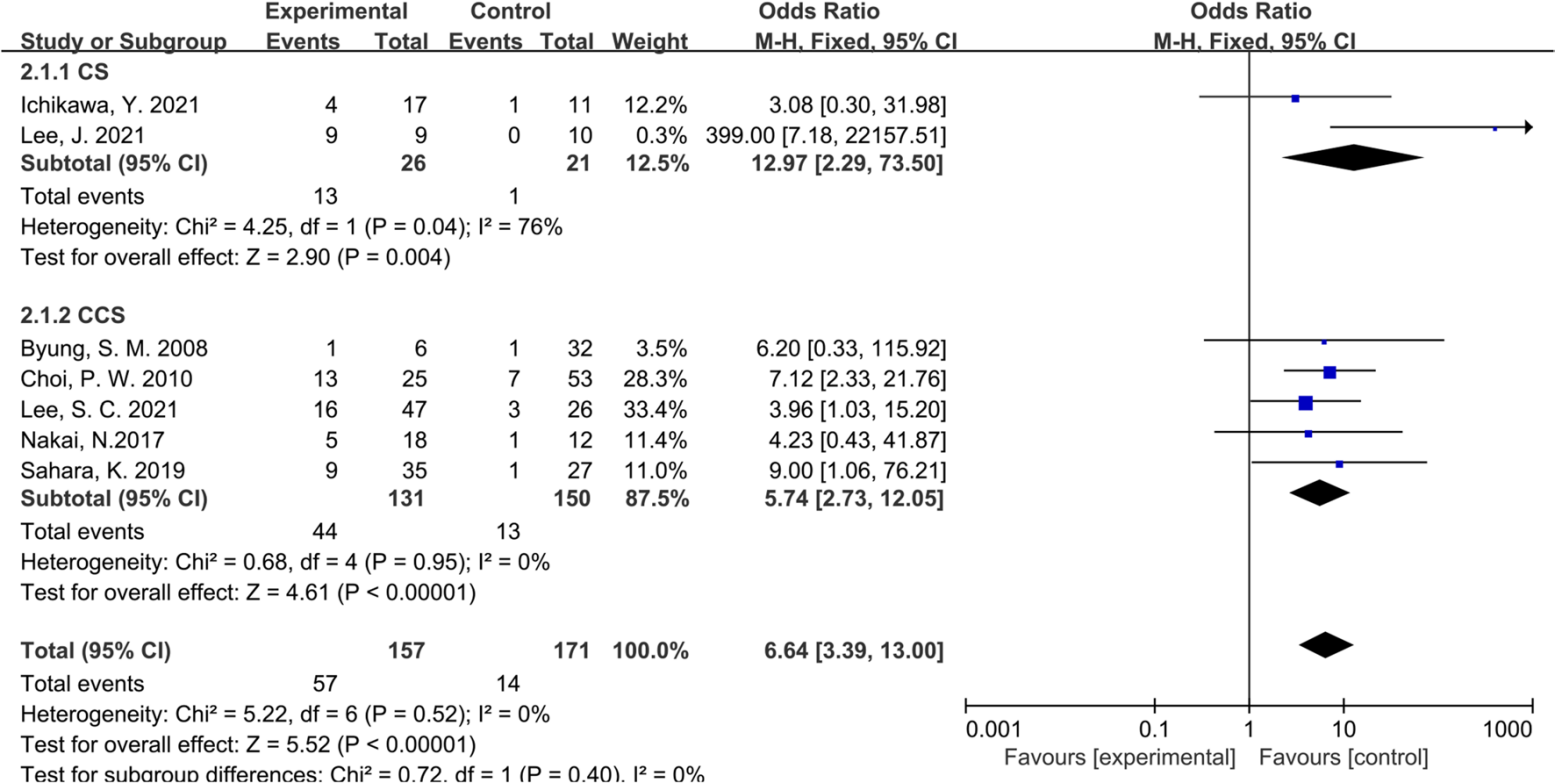
Forest plot of subgroup analysis of study types

**Fig. 5:**
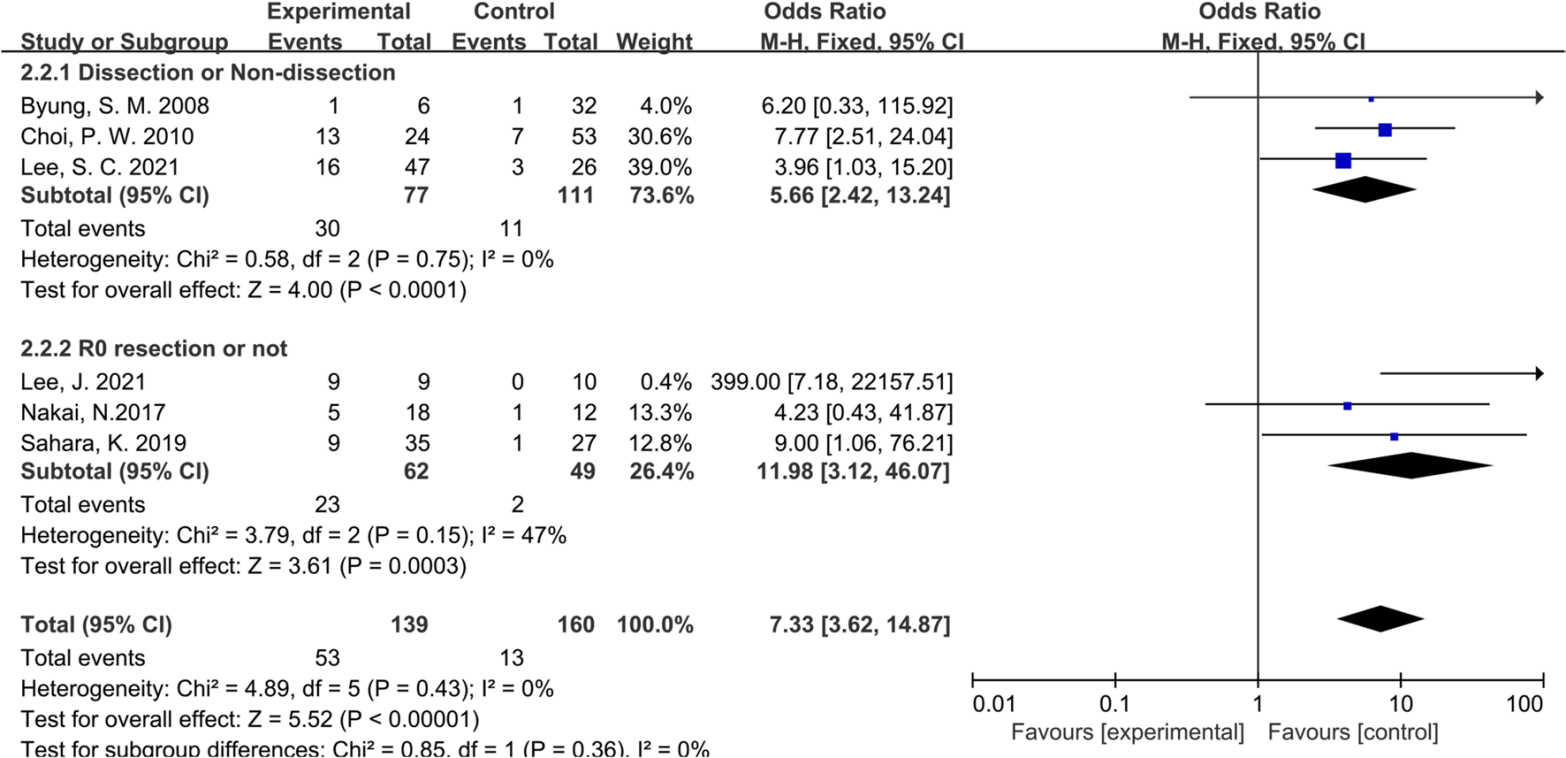
Forest plot of subgroup analysis of interventions

**Fig. 6:**
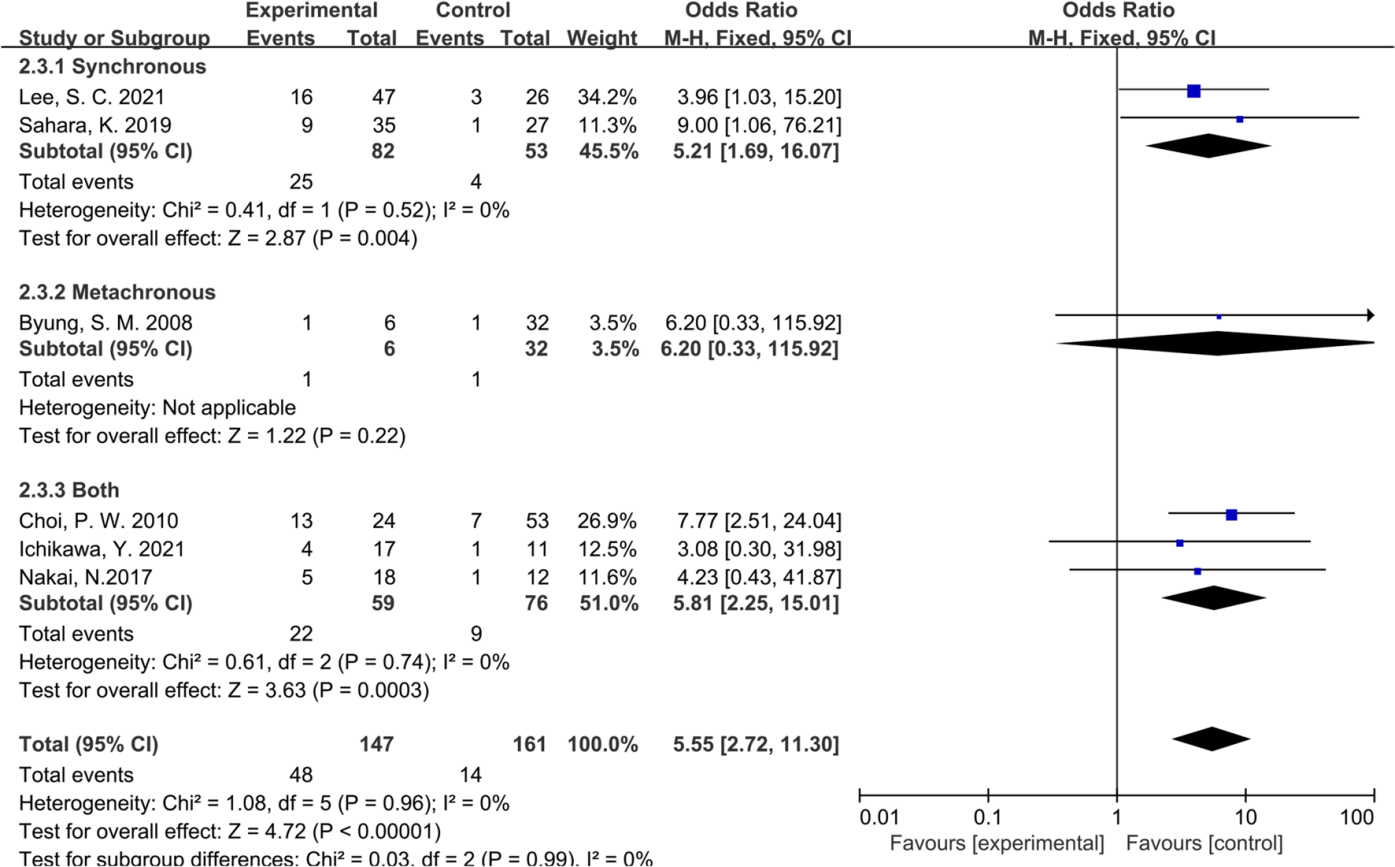
Forest plot of subgroup analysis of timing of metastasis

**Fig. 7:**
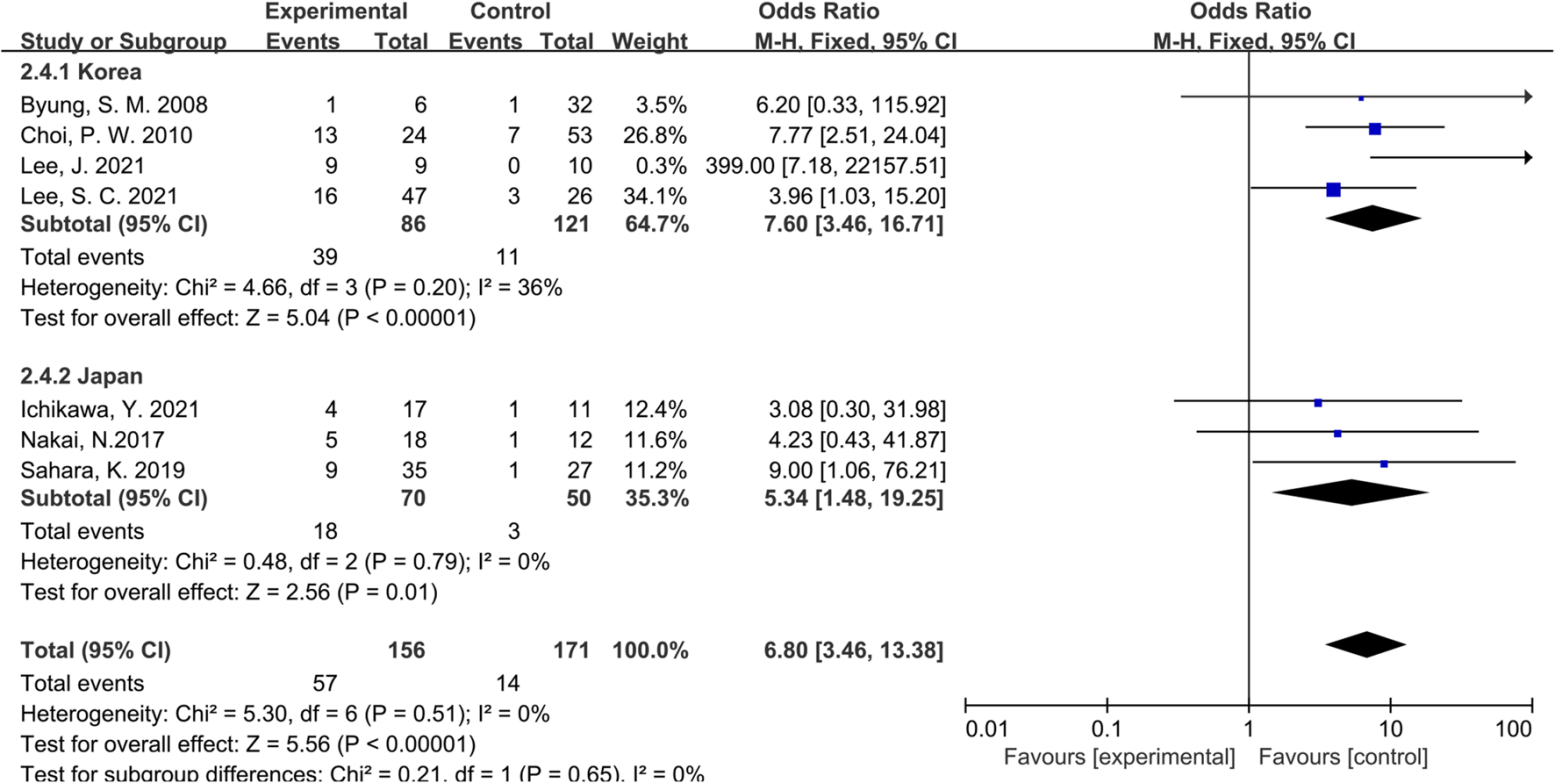
Forest plot of subgroup analysis of nationality of first author

**Fig. 8:**
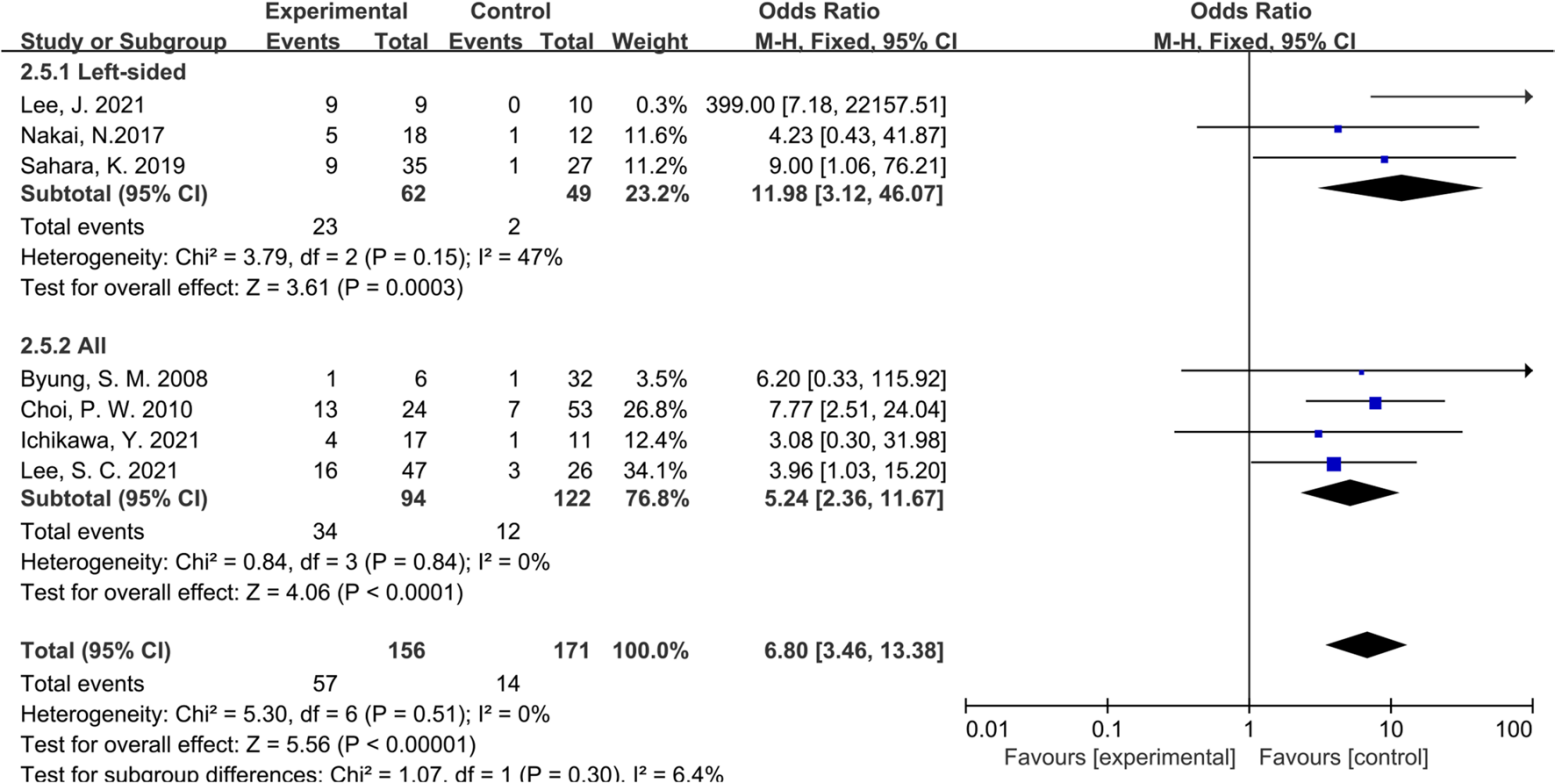
Forest plot of subgroup analysis of tumor locations

Considering that all the studies included in this paper were retrospective studies, it was necessary to conduct subgroup analysis according to different characteristics of studies. We first conducted subgroup analysis according to the study type, namely CS or CCS studies, and the results showed that the inconsistency of study types did not change the conclusions (OR: 6.64; 95% CI:3.39–13.00; P = 0.40; I^2^ = 0%), indicating that heterogeneity among the included studies was insignificant (Fig. 4). Then we performed subgroup analysis according to the management strategy. Three studies were grouped since the patients in these studies were divided according to whether PALNM resection was performed, while the remaining 4 studies were grouped for the patients were divided according to whether R0 resection was applied. Similarly, no significant heterogeneity was found between groups (OR: 7.33; 95% CI:3.62–14.87; P = 0.36; I^2^ = 0%), showing that our conclusions were relatively stable (Fig. 5). As shown in Fig. 6, the same conclusions were obtained when subgroup analysis was applied according to the time of tumor metastasis, indicating that the patients with synchronous or metachronous metastasis both benefited from radical lymphadenectomy (OR: 5.55; 95% CI:2.72–11.30; P = 0.99; I^2^ = 0%). Considering the inconformity of the staging of CRC patients with PALNM in different countries or regions, we performed subgroup analysis according to the nation. As a result, we found that the classification by country did not overturn the conclusion we have drawn before (OR: 6.80; 95% CI:3.46–13.38; P = 0.65; I^2^ = 0%) (Fig. 7). Finally, we conducted a subgroup analysis based on the location of primary tumor, and 3 studies involving 111 patients were accordingly grouped because the patients in these studies all suffered left-sided colon tumors. Although there was a certain heterogeneity within the group, it did not affect the conclusion (OR: 6.80; 95% CI:3.46–13.38; P = 0.30; I^2^ = 0%) (Fig. 8). The detailed information of subgroup analyses is presented in Table 2.

**Table 2:** Subgroup analysis and sensitivity analyses on primary outcomes

Subgroup	No. of studies	No. of patients	OR	95%CI	I^2^	P-value
*Type of studies*						
CS	2	46	12.97	2.29–73.50	76%	0.004
CCS	5	281	5.74	2.73–12.05	0%	< 0.001
*Intervention*						
Dissection or non-dissection	3	188	5.66	2.42–13.24	0%	< 0.001
R0 resection or not	3	111	11.98	3.12–46.07	47%	< 0.001
*Synchronous or metachronous*						
Synchronous	2	135	5.21	1.69–16.07	0%	0.004
Metachronous	1	38	6.20	0.33–115.92	NA	NA
Both	3	135	5.81	2.25–15.01	0%	0.0003
*Country*						
Korea	4	207	7.60	3.46–16.71	36%	< 0.001
Japan	3	120	5.34	1.48–19.25	0%	< 0.001
*Location of tumor*						
Left-sided	3	111	11.98	3.12–46.07	47%	0.0003
All	4	216	5.24	2.36–11.67	0%	< 0.001

*CS* cohort study, *CCS* case–control study, *OS* overall survival, *OR* risk ratio, *CI* confidence interval

### Quality assessment and publication bias

**Fig. 9:**
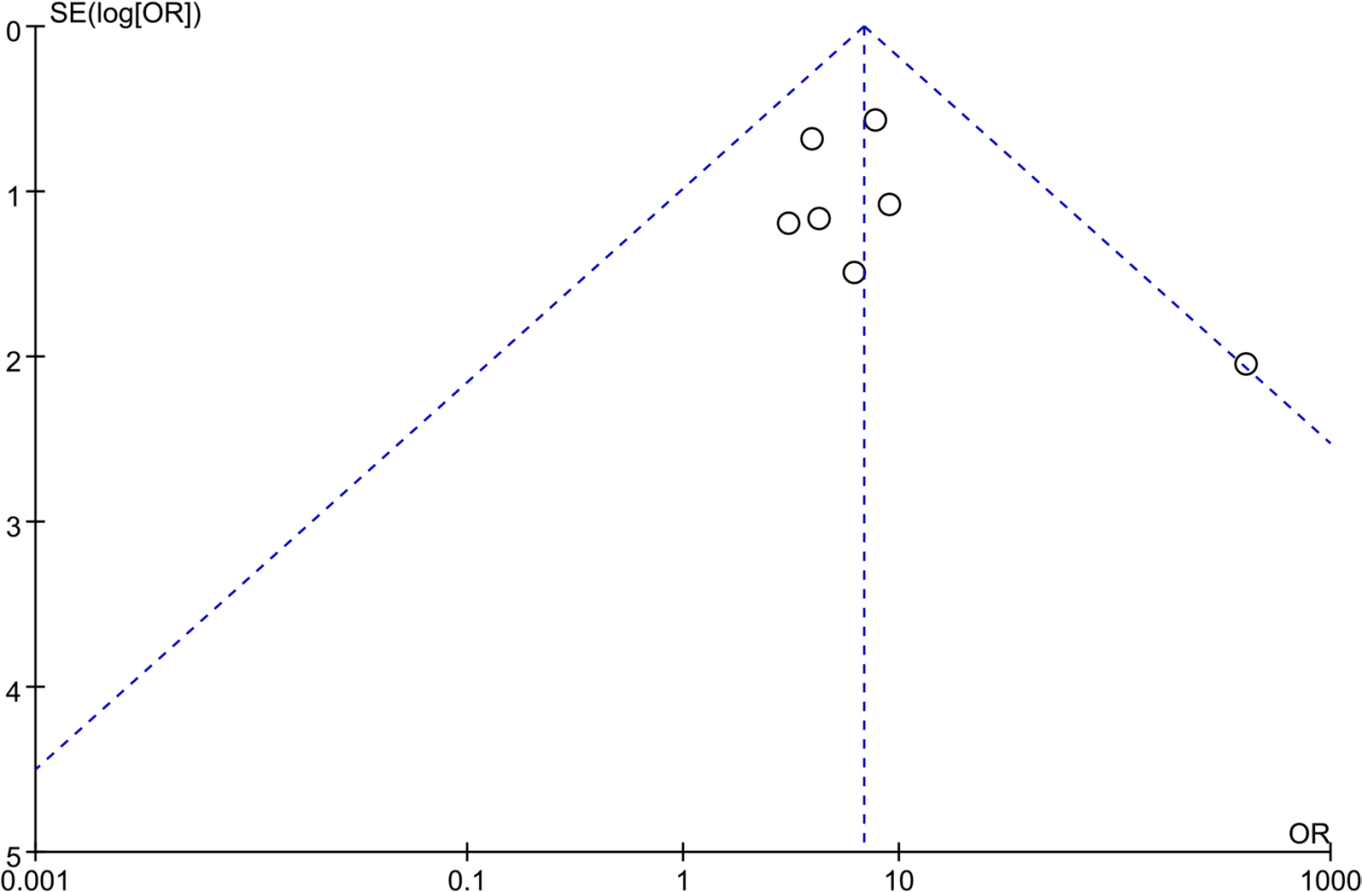
Funnel plot of all included clinical studies

According to ROBINS-I scoring tool, all the included studies in current meta-analysis were judged to be of low or moderate risk of bias (Additional file 1: Table S1 and Additional file 1: Table S2). Moreover, a funnel plot was constructed to assess the possible publication bias of primary outcome (Fig. 9). The results indicated that there appeared to be no publication bias by visual inspection.

## Discussion

Para-aortic lymph node involvement in CRC is rare, with a reported incidence of less than 2% [13, 14]. Unfortunately, the prognosis of CRC patients with PALNM is extremely poor. Therefore, a standardized treatment regimen worldwide is essential to improve the long-term survival of these patients. Currently, the efficacy of surgical resection and treatment of CRC patients with PALNM are still controversial. In this systematic review and meta-analysis, we evaluated the clinical efficacy and safety of radical lymphadenectomy in these patients. We were pleasant to find that radical lymphadenectomy could significantly improve the 5-year OS of CRC patients with PALNM. Moreover, we observed that radical lymphadenectomy had no additional effect on the incidence of postoperative complications compared with the control group. In order to explore the stability of the conclusion, we performed subgroup analysis according to various characteristics of eligible studies although these studies did not show significant heterogeneity. The subgroup analysis showed that the conclusions we had previously reached were quite stable, whether it was based on the type of study, the treatment strategy, the time of lymph node metastasis, or the nationality of the investigator and the location of the primary tumors.

In the past few decades, there have been several retrospective cohort studies and case–control studies [15–19] conducted to explore the effectiveness of radical lymphadenectomy in improving prognosis of CRC patients with PALNM. However, these studies had limited evidence since most of them were retrospective studies with small samples. To date, no prospective RCTs have been reported on the clinical efficacy of radical lymphadenectomy in CRC patients with PALNM. To the best of our knowledge, this is the first meta-analysis to summarize previous relevant studies and further evaluate the clinical efficacy and safety of radical lymphadenectomy in targeted patients. In 2016, Wong et al. [14] conducted a similar systematic review that included 18 studies and found that PALN dissection for isolated PALNM from CRC may confer a survival advantage in these patients. However, this study only analyzed the prognostic outcomes qualitatively and lacked quantitative results. As a result, the heterogeneity of included studies could not be eliminated.

Two studies included in this meta-analysis were cohort studies, and one was conducted by Lee [20], which included 263 patients with left colon or rectal cancer who underwent para-aortic lymph node dissection (PALND). A total of 19 (7.2%) patients with PALNM confirmed by routine postoperative pathology, of whom 9 underwent R0 resection and 10 underwent R2 resection. The survival analysis showed that patients who underwent R0 resection had a significantly longer 5-year OS than the control group (90.0% *vs.* 0.0%; P = 0.014). Another cohort study [21] included in this analysis enrolled 28 patients with pathologically confirmed PALNM from a cohort of 2910 patients with primary colorectal cancer. The researchers chose 3-year RFS as the primary endpoint and reached a similar conclusion that radical lymphadenectomy could improve survival outcomes of CRC patients with PALNM.

Lymph node (LN) metastasis is an important prognostic factor of CRC [22]. At present, several surgical methods have been developed to improve the survival rate of CRC patients and radical lymph node dissection is a very representative one, which has been the standard management for CRC surgery. Complete resection of metastatic tumors in CRC patients is known to improve survival and aggressive surgical approaches are advocated for specific patients with respectable liver and/or lung metastases [3]. However, the optimal treatment for CRC with PALNM has not been clearly defined. Although PALNM was classified as stage III in previous version, the JSCCR updated it as stage IV in the latest version of the guidelines. This shift also indicated that the medical community was constantly perfecting its views and treatment strategies of PALNM [6, 7]. Considering the discrepancies of PALNM disposal schemes in various countries, we therefore conducted a subgroup analysis according to the publishing nations. Three studies from Japan were included in the analysis, 2 of which were published before the latest edition of the guidelines was promulgated. However, the results showed no significant heterogeneity within the group (OR:5.34; 95% CI:1.48 − 19.25; P = 0.79; I^2^ = 0%) and also did not affect the final conclusion of our study.

Simultaneous and metachronous CRC metastases usually exhibit diverse biological characteristics, as reported in previous studies [23, 24]. Simultaneous metastases usually have a more aggressive clinical course and the prognosis is worse than that of metachronous metastases [25, 26]. As for the location of colon tumors, numerous studies have also confirmed that there are significant differences in the pathological features and patients’ prognosis between left-sided colon cancers and right-sided ones [4, 27, 28]. Therefore, subgroup analyses were accordingly performed based on the timing of metastasis and primary site of tumors. As a result, no significant heterogeneity was observed between and within groups, indicating that the conclusions we achieved had great consistency and stability.

### Limitations

However, several limitations may exist in this systematic review and meta-analysis. First, all the studies included in the analysis were retrospective studies, and no relevant RCT_S_ had been published by the time of screening. As the study type with the highest level of evidence in evidence-based medicine, the lack of RCTs will inevitably affect the reliability of our research conclusions. Second, the final eligible studies were all conducted in Japan or South Korea. As a global disease, CRC urgently needs joint efforts of scholars worldwide to provide more research data and strive to improve the prognosis of CRC patients with PALNM. Next, the managements in the control groups were also uneven, ranging from surveillance, systematic chemotherapy to R2 or R3 surgical resection, and all of which might become a confounding factor affecting the robustness of our conclusions. Moreover, several essential information like whether patients received neoadjuvant chemotherapy, detailed postoperative chemotherapy regimens and cycles were not mentioned in some studies. Finally, the clinical outcome indicators of included studies were relatively few. In addition to the primary outcome overall survival and incidence of postoperative adverse reactions, other secondary endpoints reflecting long-term survival of patients, such as tumor-free survival, relapse-free survival and tumor recurrence rate, were not recorded. Comprehensive analysis of these outcome indicators may be more accurate and reasonable for evaluating the survival benefit of patients.

## Conclusion

Considering the lack of RCTs in CRC with PALNM research field, small sample size and insufficient demonstration level of retrospective clinical studies, we conducted the first meta-analysis in this field, hoping to provide some reference for gastrointestinal surgeons. The radical lymphadenectomy treatment has showed the expected clinical efficacy in improving overall survival of CRC patients with PALNM. Moreover, the preemptive radical lymphadenectomy could not cause additional postoperative complications.

## Supplementary Information

**Additional file 1: Table S1.** The risk of bias domains (ROBINS-I) of included studies.

**Additional file 2: Table S2.** The Risk Of Bias In Non-randomized Studies-of Interventions (ROBINS-I) assessment tool.

## Data Availability

All the data analyzed in this study are obtained from the original articles.

## Abbreviations

CRC: Colorectal cancer
PALNM: Para-aortic lymph node metastasis
JSCCR: Japanese Society for Cancer of the Colon and Rectum
RCT: Randomized controlled trials
MeSH: Exploded medical subject heading
PRISMA: Preferred Reporting Items for Systematic Reviews and Meta-Analysis
ROBINS-I: Risk of Bias in Non-randomized Studies-of Interventions
OS: Overall survival
OR: Odds ratios
CI: Confidence intervals
RFS: Recurrence‐free survival
CS: Cohort study
CCS: Case control study
PALND: Para-aortic lymph node dissection
LN: Lymph node
