# Supplementary material for: Effect of radical lymphadenectomy in colorectal cancer with para-aortic lymph node metastasis: a systematic review and meta-analysis
Source: BMC Surg. 2022 May 14;22:181. doi: 10.1186/s12893-022-01631-x (PMC9107112; doi:10.1186/s12893-022-01631-x)
Supplement: Supplementary file 1 — Additional file 1: Table S1. The risk of bias domains (ROBINS-I) of included studies. [file 12893_2022_1631_MOESM1_ESM.docx]

**Supplemental Table S1. The Risk of bias domains (ROBINS-I) of included studies**

| **Study** | **Risk of bias domains -ROBINS-I** | | | | | | | | |
| --- | --- | --- | --- | --- | --- | --- | --- | --- | --- |
|  | **Bias due to confounding** | **Bias in selection of participants into the study** | **Bias in classification of interventions** | **Bias due to deviations from intended interventions** | **Bias due to missing data** | **Bias in measurement of outcomes** | **Bias in the selection of the reported results** | **Overall risk of bias** | |
| **Lee, S. C. et al, 2021** | Moderate | Low | Low | Moderate | NI | Low | Low | | Moderate |
| **Lee, J. et al, 2021** | Moderate | Low | Low | Moderate | NI | Low | Low | | Moderate |
| **Ichikawa, Y. et al, 2021** | Low | Low | Low | Low | NI | Low | Low | | Low |
| **Sahara, K. et al, 2019** | Moderate | Low | Low | Moderate | NI | Low | Low | | Moderate |
| **Nakai, N. et al, 2017** | Moderate | Moderate | Low | Moderate | NI | Moderate | Moderate | | Moderate |
| **Choi, P. W. et al, 2010** | Low | Low | Low | Moderate | NI | Low | Low | | Moderate |
| **Byung, S. M. et al, 2008** | Moderate | Moderate | Low | Moderate | NI | Moderate | Moderate | | Moderate |

Low, comparable with a well-performed RCT; Moderate, sound for a non-randomized study but not comparable with a well-performed RCT; Serious, important problems in this domain; Critical, too problematic to provide any useful evidence on the effects of the intervention. NI = no information. RCT = randomized controlled trial.
