# Supplementary material for: Effect of radical lymphadenectomy in colorectal cancer with para-aortic lymph node metastasis: a systematic review and meta-analysis
Source: BMC Surg. 2022 May 14;22:181. doi: 10.1186/s12893-022-01631-x (PMC9107112; doi:10.1186/s12893-022-01631-x)
Supplement: Supplementary file 2 — Additional file 2: Table S2. The Risk Of Bias In Non-randomized Studies-of Interventions (ROBINS-I) assessment tool. [file 12893_2022_1631_MOESM2_ESM.pdf]

Supplemental Table S2. The Risk Of Bias In Non-randomized Studies – of Interventions (ROBINS-I) assessment tool

ROBINS-I tool (Stage I): At protocol stage

Specify the review question

Participants	
Experimental intervention	
Comparator	
Outcomes	

List the confounding domains relevant to all or most studies

List co-interventions that could be different between intervention groups and that could impact on outcomes

ROBINS-I tool (Stage II): For each study

Specify a target randomized trial specific to the study

Design	Individually randomized / Cluster randomized / Matched (e.g. cross-over)
Participants	
Experimental intervention	
Comparator	

Is your aim for this study…?

☐ to assess the effect of *assignment to* intervention

☐ to assess the effect of *starting and adhering to* intervention

Specify the outcome

Specify which outcome is being assessed for risk of bias (typically from among those earmarked for the Summary of Findings table). Specify whether this is a proposed benefit or harm of intervention.

Specify the numerical result being assessed

In case of multiple alternative analyses being presented, specify the numeric result (e.g. RR = 1.52 (95% CI 0.83 to 2.77) and/or a reference (e.g. to a table, figure or paragraph) that uniquely defines the result being assessed.

Preliminary consideration of confounders

Complete a row for each important confounding domain (i) listed in the review protocol; and (ii) relevant to the setting of this particular study, or which the study authors identified as potentially important.

"Important" confounding domains are those for which, in the context of this study, adjustment is expected to lead to a clinically important change in the estimated effect of the intervention. "Validity" refers to whether the confounding variable or variables fully measure the domain, while "reliability" refers to the precision of the measurement (more measurement error means less reliability).

**(i) Confounding domains listed in the review protocol**				
Confounding domain	Measured variable(s)	Is there evidence that controlling for this variable was unnecessary?*	Is the confounding domain measured validly and reliably by this variable (or these variables)?	OPTIONAL: Is failure to adjust for this variable (alone) expected to favour the experimental intervention or the comparator?
			Yes / No / No information	Favour experimental / Favour comparator / No information

**(ii) Additional confounding domains relevant to the setting of this particular study, or which the study authors identified as important**				
Confounding domain	Measured variable(s)	Is there evidence that controlling for this variable was unnecessary?*	Is the confounding domain measured validly and reliably by this variable (or these variables)?	OPTIONAL: Is failure to adjust for this variable (alone) expected to favour the experimental intervention or the comparator?
			Yes / No / No information	Favour experimental / Favour comparator / No information

* In the context of a particular study, variables can be demonstrated not to be confounders and so not included in the analysis: (a) if they are not predictive of the outcome; (b) if they are not predictive of intervention; or (c) because adjustment makes no or minimal difference to the estimated effect of the primary parameter. Note that "no statistically significant association" is not the same as "not predictive".

Preliminary consideration of co-interventions

Complete a row for each important co-intervention (i) listed in the review protocol; and (ii) relevant to the setting of this particular study, or which the study authors identified as important.

"Important" co-interventions are those for which, in the context of this study, adjustment is expected to lead to a clinically important change in the estimated effect of the intervention.

**(i) Co-interventions listed in the review protocol**		
Co-intervention	Is there evidence that controlling for this co-intervention was unnecessary (e.g. because it was not administered)?	Is presence of this co-intervention likely to favour outcomes in the experimental intervention or the comparator
		Favour experimental / Favour comparator / No information
		Favour experimental / Favour comparator / No information
		Favour experimental / Favour comparator / No information

**(ii) Additional co-interventions relevant to the setting of this particular study, or which the study authors identified as important**		
Co-intervention	Is there evidence that controlling for this co-intervention was unnecessary (e.g. because it was not administered)?	Is presence of this co-intervention likely to favour outcomes in the experimental intervention or the comparator
		Favour experimental / Favour comparator / No information
		Favour experimental / Favour comparator / No information
		Favour experimental / Favour comparator / No information

Risk of bias assessment

Responses underlined in green are potential markers for low risk of bias, and responses in red are potential markers for a risk of bias. Where questions relate only to sign posts to other questions, no formatting is used.

Bias domain	Signalling questions	Elaboration	Response options
**Bias due to confounding**			
	1.1 Is there potential for confounding of the effect of intervention in this study?<br>**If N/PN to 1.1:** the study can be considered to be at low risk of bias due to confounding and no further signalling questions need be considered	In rare situations, such as when studying harms that are very unlikely to be related to factors that influence treatment decisions, no confounding is expected and the study can be considered to be at low risk of bias due to confounding, equivalent to a fully randomized trial. There is no NI (No information) option for this signalling question.	Y / PY / PN / N
	**If Y/PY to 1.1**: determine whether there is a need to assess time-varying confounding:		
	1.2. Was the analysis based on splitting participants' follow up time according to intervention received?<br>**If N/PN**, answer questions relating to baseline confounding (1.4 to 1.6)<br>**If Y/PY**, go to question 1.3.	If participants could switch between intervention groups then associations between intervention and outcome may be biased by time-varying confounding. This occurs when prognostic factors influence switches between intended interventions.	NA / Y / PY / PN / N / NI
	1.3. Were intervention discontinuations or switches likely to be related to factors that are prognostic for the outcome?<br>**If N/PN**, answer questions relating to baseline confounding (1.4 to 1.6)<br>**If Y/PY**, answer questions relating to both baseline and time-varying confounding (1.7 and 1.8)	If intervention switches are unrelated to the outcome, for example when the outcome is an unexpected harm, then time-varying confounding will not be present and only control for baseline confounding is required.	NA / Y / PY / PN / N / NI

	**Questions relating to baseline confounding only**		
	1.4. Did the authors use an appropriate analysis method that controlled for all the important confounding domains?	Appropriate methods to control for measured confounders include stratification, regression, matching, standardization, and inverse probability weighting. They may control for individual variables or for the estimated propensity score. Inverse probability weighting is based on a function of the propensity score. Each method depends on the assumption that there is no unmeasured or residual confounding.	NA / Y / PY / PN / N / NI
	1.5. **If Y/PY to 1.4**: Were confounding domains that were controlled for measured validly and reliably by the variables available in this study?	Appropriate control of confounding requires that the variables adjusted for are valid and reliable measures of the confounding domains. For some topics, a list of valid and reliable measures of confounding domains will be specified in the review protocol but for others such a list may not be available. Study authors may cite references to support the use of a particular measure. If authors control for confounding variables with no indication of their validity or reliability pay attention to the subjectivity of the measure. Subjective measures (e.g. based on self-report) may have lower validity and reliability than objective measures such as lab findings.	NA / Y / PY / PN / N / NI
	1.6. Did the authors control for any post-intervention variables that could have been affected by the intervention?	Controlling for post-intervention variables that are affected by intervention is not appropriate. Controlling for mediating variables estimates the direct effect of intervention and may introduce bias. Controlling for common effects of intervention and outcome introduces bias.	NA / Y / PY / PN / N / NI
	**Questions relating to baseline and time-varying confounding**		
	1.7. Did the authors use an appropriate analysis method that controlled for all the important confounding domains and for time-varying confounding?	Adjustment for time-varying confounding is necessary to estimate the effect of starting and adhering to intervention, in both randomized trials and NRSI. Appropriate methods include those based on inverse probability weighting. Standard regression models that include time-updated confounders may be problematic if time-varying confounding is present.	NA / Y / PY / PN / N / NI
	1.8. **If Y/PY to 1.7**: Were confounding domains that were controlled for measured validly and reliably by the variables available in this study?	See 1.5 above.	NA / Y / PY / PN / N / NI

	**Risk of bias judgement**	See Table B	Low / Moderate / Serious / Critical / NI
	Optional: What is the predicted direction of bias due to confounding?	Can the true effect estimate be predicted to be greater or less than the estimated effect in the study because one or more of the important confounding domains was not controlled for? Answering this question will be based on expert knowledge and results in other studies and therefore can only be completed after all of the studies in the body of evidence have been reviewed. Consider the potential effect of each of the unmeasured domains and whether all important confounding domains not controlled for in the analysis would be likely to change the estimate in the same direction, or if one important confounding domain that was not controlled for in the analysis is likely to have a dominant impact.	Favours experimental / Favours comparator / Unpredictable

Bias in selection of participants into the study			
	2.1. Was selection of participants into the study (or into the analysis) based on participant characteristics observed after the start of intervention?<br>**If N/PN to 2.1:** go to 2.4	This domain is concerned only with selection into the study based on participant characteristics observed *after* the start of intervention. Selection based on characteristics observed *before* the start of intervention can be addressed by controlling for imbalances between experimental intervention and comparator groups in baseline characteristics that are prognostic for the outcome (baseline confounding).	Y / PY / PN / N / NI
	2.2. **If Y/PY to 2.1**: Were the post-intervention variables that influenced selection likely to be associated with intervention?	Selection bias occurs when selection is related to an effect of either intervention or a cause of intervention **and** an effect of either the outcome or a cause of the outcome. Therefore, the result is at risk of selection bias if selection into the study is related to both the intervention and the outcome.	NA / Y / PY / PN / N / NI
	2.3 **If Y/PY to 2.2**: Were the post-intervention variables that influenced selection likely to be influenced by the outcome or a cause of the outcome?		NA / Y / PY / PN / N / NI
	2.4. Do start of follow-up and start of intervention coincide for most participants?	If participants are not followed from the start of the intervention then a period of follow up has been excluded, and individuals who experienced the outcome soon after intervention will be missing from analyses. This problem may occur when prevalent, rather than new (incident), users of the intervention are included in analyses.	Y / PY / PN / N / NI
	2.5. **If Y/PY to 2.2 and 2.3, or N/PN to 2.4**: Were adjustment techniques used that are likely to correct for the presence of selection biases?	It is in principle possible to correct for selection biases, for example by using inverse probability weights to create a pseudo-population in which the selection bias has been removed, or by modelling the distributions of the missing participants or follow up times and outcome events and including them using missing data methodology. However such methods are rarely used and the answer to this question will usually be "No".	NA / Y / PY / PN / N / NI
	**Risk of bias judgement**	See Table B	Low / Moderate / Serious / Critical / NI
	Optional: What is the predicted direction of bias due to selection of participants into the study?	If the likely direction of bias can be predicted, it is helpful to state this. The direction might be characterized either as being towards (or away from) the null, or as being in favour of one of the interventions.	Favours experimental / Favours comparator / Towards null /Away from null / Unpredictable

Bias in classification of interventions			
	3.1 Were intervention groups clearly defined?	A pre-requisite for an appropriate comparison of interventions is that the interventions are well defined. Ambiguity in the definition may lead to bias in the classification of participants. For individual-level interventions, criteria for considering individuals to have received each intervention should be clear and explicit, covering issues such as type, setting, dose, frequency, intensity and/or timing of intervention. For population-level interventions (e.g. measures to control air pollution), the question relates to whether the population is clearly defined, and the answer is likely to be 'Yes'.	Y / PY / PN / N / NI
	3.2 Was the information used to define intervention groups recorded at the start of the intervention?	In general, if information about interventions received is available from sources that could not have been affected by subsequent outcomes, then differential misclassification of intervention status is unlikely. Collection of the information at the time of the intervention makes it easier to avoid such misclassification. For population-level interventions (e.g. measures to control air pollution), the answer to this question is likely to be 'Yes'.	Y / PY / PN / N / NI
	3.3 Could classification of intervention status have been affected by knowledge of the outcome or risk of the outcome?	Collection of the information at the time of the intervention may not be sufficient to avoid bias. The way in which the data are collected for the purposes of the NRSI should also avoid misclassification.	Y / PY / PN / N / NI
	**Risk of bias judgement**	See Table B	Low / Moderate / Serious / Critical / NI
	Optional: What is the predicted direction of bias due to measurement of outcomes or interventions?	If the likely direction of bias can be predicted, it is helpful to state this. The direction might be characterized either as being towards (or away from) the null, or as being in favour of one of the interventions.	Favours experimental / Favours comparator / Towards null /Away from null / Unpredictable

Bias due to deviations from intended interventions			
	**If your aim for this study is to assess the effect of assignment to intervention, answer questions 4.1 and 4.2**		
	4.1. Were there deviations from the intended intervention beyond what would be expected in usual practice?	Deviations that happen in usual practice following the intervention (for example, cessation of a drug intervention because of acute toxicity) are part of the intended intervention and therefore do not lead to bias in the effect of assignment to intervention.<br><br>Deviations may arise due to expectations of a difference between intervention and comparator (for example because participants feel unlucky to have been assigned to the comparator group and therefore seek the active intervention, or components of it, or other interventions). Such deviations are not part of usual practice, so may lead to biased effect estimates. However these are not expected in observational studies of individuals in routine care.	Y / PY / PN / N / NI
	4.2. **If Y/PY to 4.1**: Were these deviations from intended intervention unbalanced between groups *and* likely to have affected the outcome?	Deviations from intended interventions that do not reflect usual practice will be important if they affect the outcome, but not otherwise. Furthermore, bias will arise only if there is imbalance in the deviations across the two groups.	NA / Y / PY / PN / N / NI
	**If your aim for this study is to assess the effect of starting and adhering to intervention, answer questions 4.3 to 4.6**		
	4.3. Were important co-interventions balanced across intervention groups?	Risk of bias will be higher if unplanned co-interventions were implemented in a way that would bias the estimated effect of intervention. Co-interventions will be important if they affect the outcome, but not otherwise. Bias will arise only if there is imbalance in such co-interventions between the intervention groups. Consider the co-interventions, including any pre-specified co-interventions, that are likely to affect the outcome and to have been administered in this study. Consider whether these co-interventions are balanced between intervention groups.	Y / PY / PN / N / NI
	4.4. Was the intervention implemented successfully for most participants?	Risk of bias will be higher if the intervention was not implemented as intended by, for example, the health care professionals delivering care during the trial. Consider whether implementation of the intervention was successful for most participants.	Y / PY / PN / N / NI

	4.5. Did study participants adhere to the assigned intervention regimen?	Risk of bias will be higher if participants did not adhere to the intervention as intended. Lack of adherence includes imperfect compliance, cessation of intervention, crossovers to the comparator intervention and switches to another active intervention. Consider available information on the proportion of study participants who continued with their assigned intervention throughout follow up, and answer 'No' or 'Probably No' if this proportion is high enough to raise concerns. Answer 'Yes' for studies of interventions that are administered once, so that imperfect adherence is not possible.<br>We distinguish between analyses where follow-up time after interventions switches (including cessation of intervention) is assigned to (1) the new intervention or (2) the original intervention. (1) is addressed under time-varying confounding, and should not be considered further here.	Y / PY / PN / N / NI
	4.6. **If N/PN to 4.3, 4.4 or 4.5**: Was an appropriate analysis used to estimate the effect of starting and adhering to the intervention?	It is possible to conduct an analysis that corrects for some types of deviation from the intended intervention. Examples of appropriate analysis strategies include inverse probability weighting or instrumental variable estimation. It is possible that a paper reports such an analysis without reporting information on the deviations from intended intervention, but it would be hard to judge such an analysis to be appropriate in the absence of such information. Specialist advice may be needed to assess studies that used these approaches.<br><br>If everyone in one group received a co-intervention, adjustments cannot be made to overcome this.	NA / Y / PY / PN / N / NI
	**Risk of bias judgement**	See Table	
	Optional: What is the predicted direction of bias due to deviations from the intended interventions?	If the likely direction of bias can be predicted, it is helpful to state this. The direction might be characterized either as being towards (or away from) the null, or as being in favour of one of the interventions.	

Bias due to missing data			
	5.1 Were outcome data available for all, or nearly all, participants?	"Nearly all" should be interpreted as "enough to be confident of the findings", and a suitable proportion depends on the context. In some situations, availability of data from 95% (or possibly 90%) of the participants may be sufficient, providing that events of interest are reasonably common in both intervention groups. One aspect of this is that review authors would ideally try and locate an analysis plan for the study.	Y / PY / PN / N / NI
	5.2 Were participants excluded due to missing data on intervention status?	Missing intervention status may be a problem. This requires that the *intended* study sample is clear, which it may not be in practice.	Y / PY / PN / N / NI
	5.3 Were participants excluded due to missing data on other variables needed for the analysis?	This question relates particularly to participants excluded from the analysis because of missing information on confounders that were controlled for in the analysis.	Y / PY / PN / N / NI
	5.4 **If PN/N to 5.1, or Y/PY to 5.2 or 5.3**: Are the proportion of participants and reasons for missing data similar across interventions?	This aims to elicit whether either (i) differential proportion of missing observations or (ii) differences in reasons for missing observations could substantially impact on our ability to answer the question being addressed. "Similar" includes some minor degree of discrepancy across intervention groups as expected by chance.	NA / Y / PY / PN / N / NI
	5.5 **If PN/N to 5.1, or Y/PY to 5.2 or 5.3**: Is there evidence that results were robust to the presence of missing data?	Evidence for robustness may come from how missing data were handled in the analysis and whether sensitivity analyses were performed by the investigators, or occasionally from additional analyses performed by the systematic reviewers. It is important to assess whether assumptions employed in analyses are clear and plausible. Both content knowledge and statistical expertise will often be required for this. For instance, use of a statistical method such as multiple imputation does not guarantee an appropriate answer. Review authors should seek naïve (complete-case) analyses for comparison, and clear differences between complete-case and multiple imputation-based findings should lead to careful assessment of the validity of the methods used.	NA / Y / PY / PN / N / NI
	**Risk of bias judgement**	See Table	Low / Moderate / Serious / Critical / NI

	Optional: What is the predicted direction of bias due to missing data?	If the likely direction of bias can be predicted, it is helpful to state this. The direction might be characterized either as being towards (or away from) the null, or as being in favour of one of the interventions.	Favours experimental / Favours comparator / Towards null /Away from null / Unpredictable

Bias in measurement of outcomes			
	6.1 Could the outcome measure have been influenced by knowledge of the intervention received?	Some outcome measures involve negligible assessor judgment, e.g. all-cause mortality or non-repeatable automated laboratory assessments. Risk of bias due to measurement of these outcomes would be expected to be low.	Y / PY / PN / N / NI
	6.2 Were outcome assessors aware of the intervention received by study participants?	If outcome assessors were blinded to intervention status, the answer to this question would be 'No'. In other situations, outcome assessors may be unaware of the interventions being received by participants despite there being no active blinding by the study investigators; the answer this question would then also be 'No'. In studies where participants report their outcomes themselves, for example in a questionnaire, the outcome assessor is the study participant. In an observational study, the answer to this question will usually be 'Yes' when the participants report their outcomes themselves.	Y / PY / PN / N / NI
	6.3 Were the methods of outcome assessment comparable across intervention groups?	Comparable assessment methods (i.e. data collection) would involve the same outcome detection methods and thresholds, same time point, same definition, and same measurements.	Y / PY / PN / N / NI
	6.4 Were any systematic errors in measurement of the outcome related to intervention received?	This question refers to differential misclassification of outcomes. Systematic errors in measuring the outcome, if present, could cause bias if they are related to intervention or to a confounder of the intervention-outcome relationship. This will usually be due either to outcome assessors being aware of the intervention received or to non-comparability of outcome assessment methods, but there are examples of differential misclassification arising despite these controls being in place.	Y / PY / PN / N / NI
	**Risk of bias judgement**	See Table	Low / Moderate / Serious / Critical / NI
	Optional: What is the predicted direction of bias due to measurement of outcomes?	If the likely direction of bias can be predicted, it is helpful to state this. The direction might be characterized either as being towards (or away from) the null, or as being in favour of one of the interventions.	Favours experimental / Favours comparator / Towards null /Away from null / Unpredictable

**Bias in selection of the reported result**			
	Is the reported effect estimate likely to be selected, on the basis of the results, from...		
	7.1. ... multiple outcome *measurements* within the outcome domain?	For a specified outcome domain, it is possible to generate multiple effect estimates for different measurements. If multiple measurements were made, but only one or a subset is reported, there is a risk of selective reporting on the basis of results.	Y / PY / PN / N / NI
	7.2 ... multiple *analyses* of the intervention-outcome relationship?	Because of the limitations of using data from non-randomized studies for analyses of effectiveness (need to control confounding, substantial missing data, etc), analysts may implement different analytic methods to address these limitations. Examples include unadjusted and adjusted models; use of final value vs change from baseline vs analysis of covariance; different transformations of variables; a continuously scaled outcome converted to categorical data with different cut-points; different sets of covariates used for adjustment; and different analytic strategies for dealing with missing data. Application of such methods generates multiple estimates of the effect of the intervention versus the comparator on the outcome. If the analyst does not pre-specify the methods to be applied, and multiple estimates are generated but only one or a subset is reported, there is a risk of selective reporting on the basis of results.	Y / PY / PN / N / NI
	7.3 ... different *subgroups*?	Particularly with large cohorts often available from routine data sources, it is possible to generate multiple effect estimates for different subgroups or simply to omit varying proportions of the original cohort. If multiple estimates are generated but only one or a subset is reported, there is a risk of selective reporting on the basis of results.	Y / PY / PN / N / NI
	**Risk of bias judgement**	See Table	Low / Moderate / Serious / Critical / NI
	Optional: What is the predicted direction of bias due to selection of the reported result?	If the likely direction of bias can be predicted, it is helpful to state this. The direction might be characterized either as being towards (or away from) the null, or as being in favour of one of the interventions.	Favours experimental / Favours comparator / Towards null /Away from null / Unpredictable

**Overall bias**			
	**Risk of bias judgement**	See<br>**Table** D	Low / Moderate / Serious / Critical / NI
	Optional: What is the overall predicted direction of bias for this outcome?		Favours experimental / Favours comparator / Towards null /Away from null / Unpredictable

Table B. Reaching risk of bias judgements in ROBINS-I: pre-intervention and at-intervention domains

Judgement	Bias due to confounding	Bias in selection of participants into study	Bias in classification of interventions
Low risk of bias (the study is comparable to a well-performed randomized trial with regard to this domain)	No confounding expected.	(i) All participants who would have been eligible for the target trial were included in the study; *and* (ii) For each participant, start of follow up and start of intervention coincided.	(i) Intervention status is well defined; *and* (ii) Intervention definition is based solely on information collected at the time of intervention.
Moderate risk of bias (the study is sound for a non-randomized study with regard to this domain but cannot be considered comparable to a well-performed randomized trial)	(i) Confounding expected, all known important confounding domains appropriately measured and controlled for; *and* (ii) Reliability and validity of measurement of important domains were sufficient, such that we do not expect serious residual confounding.	(i) Selection into the study may have been related to intervention and outcome; *and* The authors used appropriate methods to adjust for the selection bias; *or* (ii) Start of follow-up and start of intervention do not coincide for all participants; *and* (a) the proportion of participants for which this was the case was too low to induce important bias; *or* (b) the authors used appropriate methods to adjust for the selection bias; *or* (c) the review authors are confident that the rate (hazard) ratio for the effect of intervention remains constant over time.	(i) Intervention status is well defined; *and* (ii) Some aspects of the assignments of intervention status were determined retrospectively.

Serious risk of bias (the study has some important problems)	(i) At least one known important domain was not appropriately measured, or not controlled for;<br>*or*<br>(ii) Reliability or validity of measurement of an important domain was low enough that we expect serious residual confounding.	(i) Selection into the study was related (but not very strongly) to intervention and outcome;<br>*and*<br>This could not be adjusted for in analyses;<br>*or*<br>(ii) Start of follow up and start of intervention do not coincide;<br>*and*<br>A potentially important amount of follow-up time is missing from analyses;<br>*and*<br>The rate ratio is not constant over time.	(i) Intervention status is not well defined;<br>*or*<br>(ii) Major aspects of the assignments of intervention status were determined in a way that could have been affected by knowledge of the outcome.
Critical risk of bias (the study is too problematic to provide any useful evidence on the effects of intervention)	(i) Confounding inherently not controllable<br>*or*<br>(ii) The use of negative controls strongly suggests unmeasured confounding.	(i) Selection into the study was very strongly related to intervention and outcome;<br>*and*<br>This could not be adjusted for in analyses;<br>*or*<br>(ii) A substantial amount of follow-up time is likely to be missing from analyses;<br>*and*<br>The rate ratio is not constant over time.	(Unusual) An extremely high amount of misclassification of intervention status, e.g. because of unusually strong recall biases.
No information on which to base a judgement about risk of bias for this domain	No information on whether confounding might be present.	No information is reported about selection of participants into the study or whether start of follow up and start of intervention coincide.	No definition of the intervention or no explanation of the source of information about intervention status is reported.

Table C. Reaching risk of bias judgements in ROBINS-I: post-intervention domains

**Judgement**	**Bias due to deviations from intended intervention**	**Bias due to missing data**	**Bias in measurement of outcomes**	**Bias in selection of the reported result**
Low risk of bias (the study is comparable to a well-performed randomized trial with regard to this domain)	**Effect of assignment to intervention:**<br>(i) Any deviations from intended intervention reflected usual practice;<br>*or*<br>(ii) Any deviations from usual practice were unlikely to impact on the outcome.<br><br>**Effect of starting and adhering to intervention:**<br>The important **co-interventions** were balanced across intervention groups, and there were no deviations from the intended interventions (in terms of **implementation or adherence**) that were likely to impact on the outcome.	(i) Data were reasonably complete;<br>*or*<br>(ii) Proportions of and reasons for missing participants were similar across intervention groups;<br>*or*<br>(iii) The analysis addressed missing data and is likely to have removed any risk of bias.	(i) The methods of outcome assessment were comparable across intervention groups;<br>*and*<br>(ii) The outcome measure was unlikely to be influenced by knowledge of the intervention received by study participants (i.e. is objective) or the outcome assessors were unaware of the intervention received by study participants;<br>*and*<br>(iii) Any error in measuring the outcome is unrelated to intervention status.	There is clear evidence (usually through examination of a pre-registered protocol or statistical analysis plan) that all reported results correspond to all intended outcomes, analyses and sub-cohorts.

<u>Moderate risk of bias</u> (the study is sound for a non-randomized study with regard to this domain but cannot be considered comparable to a well-performed randomized trial)	**Effect of assignment to intervention:**<br>There were deviations from usual practice, but their impact on the outcome is expected to be slight.<br><br>**Effect of starting and adhering to intervention:**<br>(i) There were deviations from intended intervention, but their impact on the outcome is expected to be slight.<br>*or*<br>(ii) The important co-interventions were not balanced across intervention groups, or there were deviations from the intended interventions (in terms of implementation and/or adherence) that were likely to impact on the outcome;<br>*and*<br>The analysis was appropriate to estimate the effect of starting and adhering to intervention, allowing for deviations (in terms of implementation, adherence and co-intervention) that were likely to impact on the outcome.	(i) Proportions of and reasons for missing participants differ slightly across intervention groups;<br>*and*<br>(ii) The analysis is unlikely to have removed the risk of bias arising from the missing data.	(i) The methods of outcome assessment were comparable across intervention groups;<br>*and*<br>(ii) The outcome measure is only minimally influenced by knowledge of the intervention received by study participants;<br>*and*<br>(iii) Any error in measuring the outcome is only minimally related to intervention status.	(i) The outcome measurements and analyses are consistent with an *a priori* plan; or are clearly defined and both internally and externally consistent;<br>*and*<br>(ii) There is no indication of selection of the reported analysis from among multiple analyses;<br>*and*<br>(iii) There is no indication of selection of the cohort or subgroups for analysis and reporting on the basis of the results.

Serious risk of bias (the study has some important problems)	**Effect of assignment to intervention:**<br>There were deviations from usual practice that were unbalanced between the intervention groups and likely to have affected the outcome.<br><br>**Effect of starting and adhering to intervention:**<br>(i) The important co-interventions were not balanced across intervention groups, or there were deviations from the intended interventions (in terms of implementation and/or adherence) that were likely to impact on the outcome;<br>*and*<br>(ii) The analysis was not appropriate to estimate the effect of starting and adhering to intervention, allowing for deviations (in terms of implementation, adherence and co-intervention) that were likely to impact on the outcome.	(i) Proportions of missing participants differ substantially across interventions;<br>*or*<br>Reasons for missingness differ substantially across interventions;<br>*and*<br>(ii) The analysis is unlikely to have removed the risk of bias arising from the missing data;<br>*or*<br>Missing data were addressed inappropriately in the analysis;<br>*or*<br>The nature of the missing data means that the risk of bias cannot be removed through appropriate analysis.	(i) The methods of outcome assessment were not comparable across intervention groups;<br>*or*<br>(ii) The outcome measure was subjective (i.e. vulnerable to influence by knowledge of the intervention received by study participants);<br>*and*<br>The outcome was assessed by assessors aware of the intervention received by study participants;<br>*or*<br>(iii) Error in measuring the outcome was related to intervention status.	(i) Outcomes are defined in different ways in the methods and results sections, or in different publications of the study;<br>*or*<br>(ii) There is a high risk of selective reporting from among multiple analyses;<br>*or*<br>(iii) The cohort or subgroup is selected from a larger study for analysis and appears to be reported on the basis of the results.

Critical risk of bias (the study is too problematic to provide any useful evidence on the effects of intervention)	**Effect of assignment to intervention:**<br>There were substantial deviations from usual practice that were unbalanced between the intervention groups and likely to have affected the outcome.<br><br>**Effect of starting and adhering to intervention:**<br>(i) There were substantial imbalances in important co-interventions across intervention groups, or there were substantial deviations from the intended interventions (in terms of implementation and/or adherence) that were likely to impact on the outcome;<br>*and*<br>(ii) The analysis was not appropriate to estimate the effect of starting and adhering to intervention, allowing for deviations (in terms of implementation, adherence and co-intervention) that were likely to impact on the outcome.	(i) (Unusual) There were critical differences between interventions in participants with missing data;<br>*and*<br>(ii) Missing data were not, or could not, be addressed through appropriate analysis.	The methods of outcome assessment were so different that they cannot reasonably be compared across intervention groups.	(i) There is evidence or strong suspicion of selective reporting of results;<br>*and*<br>(ii) The unreported results are likely to be substantially different from the reported results.
No information on which to base a judgement about risk of bias for this domain	No information is reported on whether there is deviation from the intended intervention.	No information is reported about missing data or the potential for data to be missing.	No information is reported about the methods of outcome assessment.	There is too little information to make a judgement (for example, if only an abstract is available for the study).

Table D. Interpretation of domain-level and overall risk of bias judgements in ROBINS-I*

Judgement	Within each domain	Across domains	Criterion
Low risk of bias	The study is comparable to a well-performed randomized trial with regard to this domain	The study is comparable to a well-performed randomized trial	The study is judged to be at **low risk of bias for all domains**.
Moderate risk of bias	The study is sound for a non-randomized study with regard to this domain but cannot be considered comparable to a well-performed randomized trial	The study provides sound evidence for a non-randomized study but cannot be considered comparable to a well-performed randomized trial	The study is judged to be at **low or moderate risk of bias for all domains**.
Serious risk of bias	the study has some important problems in this domain	The study has some important problems	The study is judged to be at **serious risk of bias** in at least one domain, but not at critical risk of bias in any domain.
Critical risk of bias	the study is too problematic in this domain to provide any useful evidence on the effects of intervention	The study is too problematic to provide any useful evidence and should not be included in any synthesis	The study is judged to be at **critical risk of bias in at least one domain.**
No information	No information on which to base a judgement about risk of bias for this domain	No information on which to base a judgement about risk of bias	There is no clear indication that the study is at serious or critical risk of bias *and* there is a lack of information in one or more key domains of bias (*a judgement is required for this*).

*Also saved as table 2 in main article.
